# “Obese Equals Lazy?” Analysis of the Association between Weight Status and Physical Activity in Children

**DOI:** 10.1155/2013/437017

**Published:** 2013-02-28

**Authors:** F. Kreuser, K. Kromeyer-Hauschild, A. Gollhofer, U. Korsten-Reck, K. Röttger

**Affiliations:** ^1^Department of Rehabilitative and Preventive Sports Medicine, University Medical Center, University of Freiburg, Hugstetter Straße 55, 79106 Freiburg, Germany; ^2^Institute of Sport and Sport Science, University of Freiburg, Schwarzwaldstraße 175, 79117 Freiburg, Germany; ^3^Institute of Human Genetics, University Hospital-Friedrich-Schiller-University Jena, Kollegiengasse 10, 07740 Jena, Germany

## Abstract

*Introduction*. Literature provides evidence that overweight children are more sedentary. To verify this generalized statement behavior patterns of overweight and nonoverweight children needs to be understood. Therefore, we investigated the distribution of sedentary and activity levels in a quantitative and qualitative way. *Methods*. Data was collected from 37 randomly selected nonoverweight and 55 overweight children. They were 8 to 11 years of age. Height and weight were measured and weight status was characterized by BMI (BMI-percentile, BMI-SDS). Daily PA (physical activity) was measured by direct accelerometry. Spare time and screen time entertainment were obtained by questionnaires. *Results*. The amount of time spent “passive” was significantly higher in overweight children, while nonoverweight children were more “active.” The multiple regression model shows a significant association between weight status (BMI-SDS) and activity parameters. Additionally, screen time entertainment was significantly related to BMI-SDS. *Conclusion*. The results support the statement that overweight children are less active than nonoverweight children. The high amount of PA seems to be an important factor to prevent overweight in children given that PA shows the highest correlation to weight status. Quantitative and qualitative measurements are needed for further analysis.

## 1. Introduction

The increasing prevalence of childhood obesity is currently one of the central public health challenges in modern societies [1, 2]. Studies have shown that overweight children and adolescents generally grow up to be overweight adults [3–5]. Therefore, obesity in childhood is an important risk-factor for obesity and subsequent chronic diseases in later life [6, 7]. In contrast an active lifestyle in childhood should lead to health benefits in adulthood and is influenced by factors that were acquired as habits in early life [8]. Several tracking studies have proven the preventive and positive effects of active behavior in childhood on later life [6, 9–11]. Furthermore, these studies revealed that sedentary behavior during childhood generally leads to a more passive lifestyle in adulthood [8, 12]. Children's sedentary time is associated with more time spent consuming media, such as watching television (TV), computer (PC) use, and playing computer games. Watching TV is the most common sedentary behavior and involves a low fitness level and negative health outcomes [11, 13–15]. Sedentary periods of more than 9 h/per day are also declared as a chronic disease risk factor, independently of the activity time [16].

Besides the focus on PA behavior, interdisciplinary trails for overweight and obese children should support them additionally in their nutritional and psychological behavior because sedentary behavior has a high effect on the energy expenditure and other metabolic processes. Exercise-orientated programs should offer these children possibilities to replace screen time entertainment by daily outdoor and sports activities. Besides interventions programs, sports associations should implement nonperformance oriented workouts simplified for overweight children. 

To create such fitted interventions and sports programs it is important to work out reasons why children become overweight and therefore activity and inactivity levels of overweight and obese children must be identified and analyzed. To assess the complexity of all activity levels and the differences between nonoverweight and overweight children, accurate measurement of sedentary behavior and PA is needed. Accelerometer measurement connected with direct observation continues to be the choice for measuring activity and inactivity in young children [17]. Objective methods like accelerometry lead to accurate measurements of the volume, frequency, intensity, and duration of activity but have no opportunity to obtain a qualitative view on active or sedentary behavior. For qualitative analysis self-report instruments as questionnaires are at present the only available method to assess PA or sedentary behavior [18]. The combination of both methods could lead to a more precise identification of the background and levels of activity and sedentary behavior. 

Thus, the aim of this study was to compare the objectively measured results of overweight and nonoverweight children in their habitual and normal daily active and inactive behavior in order to investigate the statement that overweight children are more sedentary and inactive than nonoverweight children. Additionally, we want to obtain a deeper understanding of the background of PA and causes of sedentary behavior by analyzing accelerometry and questionnaire data in a qualitative and quantitative way.

## 2. Methods

### 2.1. Study Design

Data was collected from 37 randomly selected nonoverweight children from an elementary school and 55 overweight children who were participants in FITOC (FITOC—Freiburg Intervention Trial for Obese children) and visit elementary school as well. These children were measured at the beginning of the intervention trial. All children live in the same city in the south of Germany and were 8 to 11 years of age.

FITOC is a one-year interdisciplinary intervention program for obese children focused on PA enhancement. Participants receive medical examinations, nutritional and behavioral support and attend three physical education classes a week [19].

### 2.2. Measurements

The height and weight of the study subjects were measured. Weight status was characterized by BMI. 

Children were classified as nonoverweight (<90. percentile) and overweight (>90. percentile) according to national reference BMI-percentiles from German children by Kromeyer-Hauschild [20]. The individual BMI data was converted to SD scores (BMI-SDS), using the national reference data of German children [20] to compare the weight groups. The children's parents completed a questionnaire that sought information about the relative time their child spent in various spare time activities and spent in screen-time entertainment such as watching TV, using the computer, or playing videogames. 

PA was assessed using an accelerometry-based motion sensor (AiperMotion 440, Aipermon GmbH, Germany). The system uses 3 D acceleration sensors and analyzes data with a disclosed online algorithm. Two different algorithms were used. The first classified the accelerometric data to times with (“active time”) and without PA (“passive time”) with a 4 s resolution. The second algorithm calculated the “active” acceleration rates into four activity levels (rest, low, moderate, and high) which were based on a pilot study. In the pilot study we observed the children while performing different activities. We evaluated the different activities by direct observation measuring exact acceleration rates after every activity to precisely define the intensity classes. The 4 classes were divided into different degrees of acceleration (acceleration rates).  Table 1 shows the results of the pilot study. 

Activity distribution was calculated for each child in the assessed time periods. “Non-wear-time” was determined as ≥20 minutes of consecutive nonacceleration and was excluded. For weekdays and weekend days the arithmetic mean of the relative activity was calculated. These results were displayed as minutes in the different activity level. 

Subjects were requested to wear the accelerometer on a belt at the hip for 3 weekdays (WD) and 2 weekend (WE) days. The accelerometer recorded the activity from the moment it was turned on; only while sleeping, bathing or dressing the subject removed the device. Data was collected during spring time between 7 am and 9 pm.

### 2.3. Statistical Analysis

The statistical analyses were performed using SPSS 19.01. All accelerometer data were exported to MATLAB for further analysis. Descriptive statistics were made according to anthropometrical data and physical activity time. 

Independent sample *t*-test exploring differences in physical activity times and levels between overweight and nonoverweight children were performed. Weight class and sex differences between TV time, PC use, and playing games as well as spare time items were tested by using a chi-square test for categorical variables.

A multiple regression model was used to investigate the correlation between BMI-SDS and physical activity levels as well as spare time variables.

The significance level was set at *α* = 0.05. The study was approved by the Ethics Committee of the University of Freiburg. 

## 3. Results

### 3.1. Anthropometry

Anthropometric data such as age, weight, height, and BMI-SDS of nonoverweight and overweight children were obtained. Nonoverweight children show in average a BMI-SDS value of −0.13 ± 0.57 (SD) while overweight children of 2.08 ± 0.56 (SD). Results are shown in Table 2(a). All children were in a prepuberal state according to Tanner stages [21, 22]. We did not find gender differences in both weight classes.

### 3.2. Physical Activity

#### 3.2.1. Active and Passive Times

Regarding the activity behavior of nonoverweight and overweight children, nonoverweight children are significantly more active and less passive than overweight children (Table 2(b)). Comparing active and passive times during WD and WE, significant differences were found (*P* < 0.001  and  *P* = 0.023), respectively. 

**Figure 1:**
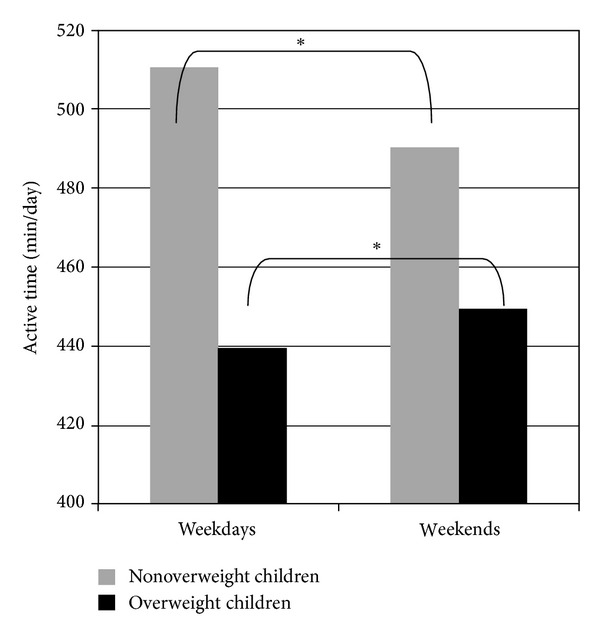
Total active time (min/day) of weight classes during weekdays and weekends. Median values were significant (*P < 001) within nonoverweight and within overweight children between weekdays and weekends.

Comparing the activity time in the weight groups, nonoverweight children are significantly less active during WE than during WD while overweight children are significantly more active during WE than during WD (*P* < 0.001) (Figure 1).

#### 3.2.2. Activity Levels

During WD nonoverweight children are highly significantly more active and less passive in all activity levels as overweight children: rest (*P* ≤ 0.001), low (*P* = 0.002), moderate (*P* ≤ 0.001), and high (*P* = 0.004). Only during WE there were no differences found in the categories “rest” (*P* = 0.08), “low” (*P* = 0.786) and “moderate” (*P* = 0.135) while in the category “high”, significant differences were found (*P* = 0.003) (Table 2(b)).

#### 3.2.3. Screen-Time Entertainment

Nonoverweight children consume highly significantly lower screen-media than overweight children during WD (*P* = 0.001) and during WE (*P* = 0.002). Overweight children use the computer significantly more often and play more computer games than their nonoverweight counterparts (*P* ≤ 0.001) (Table 2(b)). 

### 3.3. Spare Time Activity

Analyzing the questionnaire, nonoverweight children play more frequently outside in their spare time than their overweight counterparts (*P* = 0.003). 47.06% of the nonoverweight children played for more than 6 days/week outside, while only 28.13% of the overweight children did. Furthermore, 13.4% of the nonoverweight children are reported to participate in various organized individual or team sports or other organized physical training session. This was generally through a local or neighborhood association or group more than 3 days/week, while none of overweight children's parents reported any such participation (*P* = 0.003). However, comparing these two weight groups, we found having a membership in such association or groups did not show differences with being in either weight group (*P* = 0.513). Furthermore, no difference between groups was found between the frequency of engaging in individual sports or being active during the weekend (*P* = 0.769). 

Nonoverweight children spend significantly less time doing their homework (*P* = 0.04). Overweight children (35.48%) needed more than 60 minutes to complete their homework assignments, while only 11.76% of the normal weight children did (Table 2(b)).

### 3.4. Regression Models

The multiple regression model shows a significant association between weight status (BMI-SDS) and activity parameters as well as spare time behaviors. A variance of 71.2% was explained through the regression model (*R* ^2^ = 0.71). The highest influence was given by PA parameters. TV and PC consumption had also a significant influence on BMI-SDS.  Table 3 shows the results. 

For all activity and leisure time parameters there were no gender differences. 

## 4. Discussion

### 4.1. Tracking Effect and Sedentary Time

This study clearly demonstrated the association between overweight and sedentary lifestyles. It has been proven that obesity leads to greater negative health consequences in adulthood if children maintain an inactive lifestyle during childhood and adolescence [6, 23]. Passive behavior has been established as a field of research. Therefore, many papers discuss potential negative health outcomes starting their sedentary lifestyle in childhood [7, 16, 24]. 

For a further discussion of the present study we want to distinguish between sedentary time explained through a higher screen-time entertainment associated with passive time and active time explained through PA, sports club memberships or simply playing outside. While being overweight is widely discussed to have a higher correlation to sedentary rather than to active behavior, sedentary behavior must be considered as independent factor when assessing correlations to overweight [24]. 

### 4.2. Passive Time

In this study, “passive time” as well as the lowest activity level (“rest”) was associated with sedentary behavior. Furthermore, screen-time entertainment, such as watching TV, using the PC, playing video games, and doing homework was categorized as sedentary behavior. Based on all assessed sedentary time behaviors, overweight children spend significantly more time being passive than their normal weight counterparts. In general, measuring sedentary behavior presents a challenge because there are a variety of activities that are defined as “passive time”, such as TV viewing, using other screen-time entertainment, or sitting in class. Every following discussed literature uses one of the mentioned “descriptions” to define their sedentary time.

Our finding that being overweight is correlated with passive time is consistent with several studies on children [7, 16, 25–27]. Purslow et al. assessed sedentary behavior in children by accelerometry and used the lowest activity level as “sedentary”. This study found a significant association between children's fat mass index and inactivity and confirms our result that overweight children are more passive [28]. TV consumption is the most prevalent sedentary behavior for overweight children and adolescents and involves a low fitness level and negative health outcomes [8, 11, 13, 14]. Janz et al. found a strong association between TV viewing and fatness while assessing body composition in children [12] and proved the association between sedentary behavior and overweight. Only a minority of studies reported less or no difference between weight groups in their sedentary behavior such as a study from Belgium [29]. These studies did not find differences in the amount of inactive play, in the assessed light intensity or sedentary intensity measured by accelerometry, and in children's screen-time behavior during the week [30].

Furthermore, we assessed the time children spent doing their homework and a novel finding in the current study was that we found a significant difference between weight groups. Overweight children spend more time on average doing their homework than nonoverweight children. A possible explanation could be that sedentary lifestyle also influences neurocognitive function and academic performance showing children with low physical activity levels to have poorer academic achievement scores and inferior cognitive performance compared to physically fit children [31–33]. 

Nearly all studies mentioned indicate that sedentary behavior, independent of levels of active behavior, show negative health outcomes during childhood. Since sedentary behavior is omnipresent, Canadian Sedentary Guidelines were created in 2011 for the enhancement of children's health [34]. This is one first step to reduce sedentary behavior and to promote an active lifestyle to decrease the prevalence of childhood obesity.

### 4.3. Active Time

Evaluating the active time, our data demonstrates a significant difference between weight classes. In this study active behavior is characterized by accelerometer-based PA levels, playing outdoors, and spending time in sports associations. Nonoverweight children are significantly more active in all activity levels than overweight children. 

Correlations between sedentary time and weight status have been discussed above, while we presently focus on studies integrating activity in different levels and overweight. 

Screening literature, Deforche et al. reported that nonoverweight children spend more time on average in moderate-to-vigorous physical activity (MVPA) than overweight children [30]. Colley et al. confirm that overweight boys accumulate less MVPA than their nonoverweight counterparts, but this difference was not found in girls [35]. Studies from the United States and Australia found significant inverse linear relationships between PA and both BMI and body fat [36, 37]. One large study from Europe examined the association between PA measured by Actigraph and body composition. Although they did find a correlation between MVPA and body fatness in children, this relation was very weak [38]. Ness et al. objectively measured PA by accelerometry and presented a strong inverse relationship between obesity and MVPA. They compared PA with BMI and body composition measured by DXA scanner [39]. In our multiple regression model, BMI-SDS and time being physically active showed the highest association and a strong inverse correlation between activity and obesity as well. 

### 4.4. Activity Levels during Weekend

Treuth et al. focused on girls aged 11 and found out that nonoverweight children spent more time in MVPA than overweight children, but this difference was smaller on weekends than on weekdays. This fact is comparable with our results showing that nonoverweight children are significantly less active during WE than during WD while, interestingly, overweight children are significantly more active during WE than during WD. Further studies examined and confirmed a lower activity time in nonoverweight children on weekends [30, 40, 41]. This could be caused by regular activity in sports associations and clubs during WD. An explanation for a higher PA level in overweight children on WE could be their parents' spending spare time with them, because it is known that overweight children live more often in single parent families who probably work during week but not during WE [42–44]. This significant difference between WD and WE shows the need of activity support by public health, social and sports organizations, especially in overweight children. Financial encouragement for low income families for an easier accessibility in sports organizations should be implemented. Furthermore, a wide range of offers in all kind of sports should be the first step to decrease the high thresholds for overweight children to take part in sports organizations. 

### 4.5. Spare Time

We asked the parents if their children had a membership in sports associations and clubs, and about the frequency their children took part in organized sports during the week. We did not find a difference between weight groups in having a membership. However, we did find a difference in the frequency; children take part in individual or team sporting events or training sessions during the week. None of the overweight children took part in 3 or more lessons during the week. This is confirmed by an Australian study, where the authors show that overweight children were significantly less involved in community organizations [45]. Trembley and Willms found out that PA in organized and unorganized sports is negatively associated with obesity [46].

PA time is also characterized by playing outside. We observed a lower level of outdoor playing in overweight children. Only few studies examined the spare time activity, but Dolonski et al. found a significant correlation between PA (MVPA) and outdoor play [47]. Veitch et al. focused on the neighborhood social environment and children's weight and found an association between BMI and the time spent outdoors [48]. We only found one study that did not show differences in hours of outside play between overweight and nonoverweight children [30].

All these results show the need of creating interventions and programs that are highly stimulative in nature for overweight children, fitted to their interests to enhance PA. Interventions which reduce anxiety associated with PA should be implemented, and it is a basic necessity to involve overweight children in programs like FITOC. Overweight and obese children may benefit from PA-focused trials that are designed to encourage daily PA. Factors such as fun, variety, family participation, and motivational aspects can help overweight children to recover enjoyment in PA. Furthermore increased sessions to improve the development of fundamental movement skills and PA behavior should be implemented throughout school week. Additionally home activities that encourage children to be more active should help to reduce screen-based entertainment. This is a challenge for public health programs, teachers, and sports organizations.

### 4.6. Limitations

Quantifying and measuring PA in the complex behavior of children is a difficult undertaking. Children's PA is characterized as spontaneous and irregular because they have a short attention span, in contrast to a consistently distributed PA in adults. Several studies used for children's PA assessment were proxy reports, diaries, or direct observation to define PA [49–51]. Proxy reports are decidedly less objective because they were filled out by parents [52]. In our study parents completed a questionnaire that sought information about the time their child spent in spare time activity and screen-time entertainment as well. To prove objectivity we compared activity times measured by accelerometry and answers given by questionnaires. For different levels of PA and sedentary time, accelerometry continues to be the method of choice. Accelerometers have the advantage to evaluate the frequency, intensity, and duration of PA over a longer time-period and are objective [53]. Although accelerometers have their limitations being not used for qualitative analysis, they have currently a great deal of utility, especially in young children [53]. 

For our study we chose children 8–11 years old, because this age group takes part in the intervention trail FITOC. The nonoverweight children were recruited in this age group too. We did not find gender differences in their activity behavior, in their spare time behavior, or in their media consumption. Therefore different gender distribution in both weight groups does not affect the results and gender differences do not have to be mentioned in this paper. 

## 5. Conclusion

This study highlights strong evidence that overweight children spend more time sedentary and accumulate significantly lower levels of PA than their nonoverweight counterparts. The problem of overweight and obesity leads to negative health outcomes and must be handled immediately with public health strategies. The present worldwide change in children's sedentary lifestyle makes it a fruitful area for further research. 

The current finding illustrates the need to establish interventions, especially weekdays, targeted to overweight children, because PA plays an integral role for immediate and long-term health implications. More proposals must be created inside sport associations for overweight children: enjoyable and doable, not performance-oriented and without competition. Furthermore, intervention research must move towards to identify how feasible intervention arrangements can be established in the education and health system to obtain long-term effective impacts. 

### 5.1. What Is Already Known about the Study?

There is a link between inactivity and overweight in children.

Overweight children have a significantly higher screen time entertainment (TV, PC) than nonoverweight children.

Nonoverweight children are more active (outdoor play, daily PA) than overweight children.

### 5.2. What Does This Study Add?

In contrast to other studies measuring PA objectively but only quantitatively, or studies exploring PA only by self-report but in a more qualitative way, this study combines PA measured by objective accelerometry and by proxy report of parents who gave additional information about PA time and spare time activity. This leads to a significant quantitative and qualitative analysis of PA and sedentary behavior.

Overweight children are in general more active on weekends and, with respect to the “moderate” activity level, as active as their nonoverweight counterparts. Thus, overweight children are not always as inactive as mentioned.

Overweight children spent more time doing their homework than nonoverweight children. That could be caused by the evidence-based fact that sedentary lifestyle also can influence and reduce neurocognitive function.

## Figures and Tables

**Table 1:** Observation of accelerometer rates during direct observation.

Level of acceleration	Kind of sports	Degree of acceleration (acceleration rate)
>60 high activity	Skipping rope	103.4
	Running/jogging	92.3
	Walking/Nordic walking	89.7
	Climbing stairs	72.2
	Basketball	71.5
	Soccer	68.2
	Playing tag	66.6

35–60 moderate activity	Hockey	59.2
	Class breaks	51
	Walk/ride to school	49.1
	Playing	49
	Inline skating	48

10–35 low activity	Playing inside	31

<10 rest	Reading/screen time	5.1
	Sitting	4.1

Values shown are expressed as median degree of acceleration. Data were taken from *n* = 20 children.

**Table:** (a)

*N* = 92	“Nonoverweight”	“Overweight”	*P* values
	*n* = 37	*n* = 55	
Anthropometry			
Age (y)	8.77 ± 0.92	9.28 ± 1.2	*P* = 0.04
Height-SDS	0.47 ± 0.95	0.99 ± 1.16	*P* = 0.26
Weight-SDS	0.13 ± 0.56	2.06 ± 0.61	*P* ≤ 0.001
BMI-SDS	−0.13 ± 0.57	2.08 ± 0.56	*P* ≤ 0.001
BMI	16.2 ± 1.2	24.6 ± 2.91	*P* ≤ 0.001
Sex (boys/girls)	♂37.84%, ♀62.16%	♂36.36 %, ♀63.64 %	

Age in years, height-SDS, weight-SDS, Body-Mass-Index-Standard Deviation Score and BMI expressed as mean ± standard deviation. Sex (boys and girls) was expressed in percentage.

**Table:** (b)

*N* = 92	“Nonoverweight”		“Overweight”	
	*n* = 37		*n* = 55	
Activity^a^ (min)	WD	WE	WD	WE

Passive time	329.5 (300; 395)**	349.7 (299; 399)	400.5 (362; 441)	390.7 (307; 468)
Active time	510.5 (445; 540)**	490.3 (441; 540)	439.5 (399; 478)	449.2 (372; 533)

Activity levels (min)				
“rest”	327 (274; 383)**	341.0 (287; 401)*	417.2 (265; 468)	390.0 (298; 457)
“low”	270.5 (236; 300)*	252.2 (202; 284)	244.6 (210; 275)	243.7 (214; 284)
“moderate”	155.5 (134; 193)**	168.5 (134; 196)	129.3 (99; 150)	144.2 (105; 194)
“high”	67.3 (42; 102)*	69.3 (42; 99)*	44.2 (26; 76)	51.0 (28; 81)

Screen-Time hours^b^	WD	WE	WD	WE
TV (>60 min/day)	38.24%*	58.82%*	80%	85.29%
PC (>60 min/day)	2.94%**	2.94%**	37.14%	40%
Games (>60 min/day)	0%**	2.94%**	23.53%	44.12%

Spare-Time activity^c^				
Homework (>60 min/day)	11.76%*		35.48%	
Sports club member	70.59%		57.58%	
Sports club (>3 days/wk)	28.0%*		0%	
Outdoor sports (>6 days/wk)	47.06%*		28.13%	

^a^WD: weekday, WE: weekend. Daily activity time in minutes expressed as median (25th percentile; 75th percentile). Differences were significant (*U*-Test) between weight classes (**P < 0.001, *P < 0.05).

^b^Screen-time entertainment (more than 60 min/day) in percent (%) for television (TV), computer (PC), and computer games (Games). Differences were significant (Chi-square test) between weight classes (**P < 0.001, *P < 0.05).

^c^Spare time activity. Homework more than 60 min/day, Sports club membership, more than 3 sports club sessions per week. Outdoor: “how often does your child play outside per week?” (more than 6 times/week). Differences were significant (Chi-square test) between weight classes (**P < 0.001, *P< 0.05).

**Table 3:** Spare time variables and accelerometer data associated with BMI-SDS.

Variables	*β*_adjusted_	*P* value
PA “WE moderate activity”	−0.955	0.001
PA “WE active time”	0.849	0.003
PA “WD high activity”	−0.639	0.000
PA “WD low activity”	−0.574	0.002
PA “WD moderate activity”	0.414	0.016
PC (WD)	0.361	0.021
PC (WE)	0.355	0.035
Age	−0.261	0.042
TV (WE)	0.256	0.017

Linear multiple regression model (*R* ^2^ = 0.71).

Excluded variables: games (weekday), homework (time/day), sports club frequency, spare time times/week, WE low activity, WE passive time, frequency sport WE, TV (WD), games (WE), WE high activity, WD active time, sports club member, leisure time activity.

PA: physical activity, WD: weekday, WE: weekend, TV: television, PC: computer, BMI-SDS: Body-Mass-Index Standard Deviation Score.
